# Short-Term Outcomes of Faricimab Treatment in Aflibercept-Refractory Eyes with Neovascular Age-Related Macular Degeneration

**DOI:** 10.3390/jcm12155145

**Published:** 2023-08-06

**Authors:** Maya Kishi, Akiko Miki, Aya Kamimura, Mina Okuda, Wataru Matsumiya, Hisanori Imai, Sentaro Kusuhara, Makoto Nakamura

**Affiliations:** Department of Surgery, Division of Ophthalmology, Kobe University Graduate School of Medicine, Kobe 650-0017, Japan; mk0204@med.kobe-u.ac.jp (M.K.);

**Keywords:** age-related macular degeneration, anti-vascular endothelial growth factor, faricimab, aflibercept

## Abstract

To evaluate the functional and anatomical effects of switching to faricimab for patients with neovascular age-related macular degeneration (nAMD) refractory to intravitreal aflibercept, this retrospective study evaluated patients with nAMD who received intravitreal injections of aflibercept (IVA) every <8 weeks and were switched to faricimab. After switching, the patients were treated with a treatment and extended regimen that started with the interval just before switching and received at least three injections. We evaluated changes in the best-corrected visual acuity (BCVA), central retinal thickness (CRT), central choroidal thickness (CCT), treatment interval, and presence of retinal fluid. Overall, 55 eyes from 55 patients were examined. After three injections of faricimab, the BCVA and CCT did not change significantly. However, the CRT decreased significantly (*p* < 0.05), the injection interval was significantly extended (7.5 ± 2.3 vs. 5.9 ± 1.5 weeks, *p* < 0.01), and the rates of the presence of intraretinal fluid and subretinal fluid decreased significantly to 16.4% and 40% of eyes, respectively (both *p* < 0.01). An ocular adverse event (retinal pigment epithelium tear) developed in one eye. Switching to faricimab was effective for anatomic changes. It may be an additional treatment option for some eyes refractory to IVA.

## 1. Introduction

Age-related macular degeneration (AMD) is a leading cause of blindness [1]. Anti-vascular endothelial growth factor (VEGF) is the gold standard therapy for favorable visual and anatomical effects [1]. However, the response to treatment varies widely among patients. Some patients fail to extend the treatment interval or have poor fluid control [2] even when treated with continuous intravitreal anti-VEGF injections. In such patients, frequent injections are required, which increases the financial and physical burden, leading to poor follow-up adherence and resulting in the deterioration of visual acuity [3]. Hence, longer-acting drugs are desirable.

Recently, faricimab was approved for neovascular AMD (nAMD). Based on the TENAYA and LUCERNE clinical trials, the change in the best-corrected visual acuity (BCVA) in eyes treated with 6 mg of faricimab was non-inferior to that in eyes treated with 2 mg of aflibercept [4]. The 48-week results of the TENAYA clinical trials showed that 34.0% and 45.7% of eyes treated with 6 mg of faricimab maintained the q12w and q16w dosing intervals, respectively. Similarly, the 48-week results of the LUCERNE clinical trials showed that 32.9% and 44.9% of eyes treated with 6 mg of faricimab were maintained on the q12w and q16w dosing intervals, respectively. These results suggest that faricimab may extend the treatment interval.

Based on these results, we hypothesized that switching to faricimab could control exudative changes and extend injection intervals in eyes that were poorly controlled with injections every 4 weeks and in refractory cases that were controlled with treatment intervals of less than 8 weeks. Few studies have reported the treatment outcomes of faricimab after switching from aflibercept. Therefore, in this study, we retrospectively evaluated the treatment outcomes of faricimab after switching from aflibercept in patients with nAMD requiring treatment every <8 weeks.

## 2. Materials and Methods

This study was approved by the institutional review board of Kobe University Hospital (No. 180290) and conducted in accordance with the tenets of the Declaration of Helsinki.

### 2.1. Study Design and Participants

We enrolled patients with nAMD who received intravitreal injections of aflibercept (IVA) every <8 weeks that were switched to faricimab by September 2022 at Kobe University Hospital and had received three injections of faricimab. We included poorly controlled non-responders with injections every 4 weeks and refractory cases that were controlled with treatment intervals of less than 8 weeks. All the patients underwent ophthalmologic examinations, including dilated fundus examination, BCVA measurement using a Landolt C chart, and slit-lamp examination, before each injection. The nAMD subtypes were diagnosed based on a previous study [5]. The Spectralis OCT system (Heidelberg Spectralis OCT; Heidelberg Engineering GmbH, Heidelberg, Germany) was used to obtain macular scans, fluorescein angiography, and indocyanine green angiography. We used a 6-line macular radial scan and a 31-line horizontal raster scan of the OCT images using enhanced depth imaging (EDI) through the fovea to evaluate any exudative changes, including intraretinal fluid (IRF) and subretinal fluid (SRF) at the fovea, and to measure the OCT parameters. Clinical data were recorded, including the OCT parameters, the presence of IRF and SRF at the fovea, and the BCVA before and after treatment. The CRT was manually measured from the outer surface of the neurosensory retina to the inner surface of the retinal pigment epithelium (RPE) at the foveal center. The central choroidal thickness (CCT) was also manually measured from the outer surface of the RPE to the inner surface of the sclera at the foveal center on the EDI-OCT images.

### 2.2. Treatment

All the participants received at least three intravitreal injections of 6.0 mg/0.05 mL of faricimab following dilated fundus examination, BCVA measurement, and OCT examination. The patients received intravitreal injections of faricimab based on the treatment and extended regimen starting from the injection interval prior to the switch and without a loading phase. The treatment intervals were then adjusted for 2 weeks based on the presence of exudative findings on the OCT. The minimum injection interval was 4 weeks.

### 2.3. Statistics Analyses

The decimal BCVA was converted into the logMAR for the statistical analyses. The BCVA and OCT measurements were analyzed using the Wilcoxon signed-rank test with the Bonferroni correction. The difference in the treatment interval before and after switching to faricimab was analyzed using the Wilcoxon signed-rank test. Fisher’s exact test with the Bonferroni correction was used to compare the proportion of eyes with IRF or SRF before and after the switch. In addition, Fisher’s exact test was used to compare the baseline CCT among the nAMD subtypes. A comparison between the patients with injection intervals of ≥8 weeks and <8 weeks was performed using Fisher’s exact test for the categorical variables and the Mann–Whitney test for the quantitative variables. A *p*-value of <0.05 was considered statistically significant. All the statistical analyses were conducted using the SPSS software version 25.0 (IBM, Armonk, NY, USA).

## 3. Results

This study included 55 eyes of 55 patients with nAMD who received IVA every <8 weeks, were switched to intravitreal injections of faricimab (IVF) and received three consecutive injections after switching. Table 1 shows the clinical characteristics of the study participants. The mean age was 80.1 ± 7.0 years, and the proportion of men was 61.8% (34 of 55 patients). The baseline BCVA was 0.26 ± 0.34 logMAR. The mean treatment interval before switching to faricimab was 5.9 ± 1.5 weeks. The baseline central retinal thickness (CRT), CCT, and pigment epithelial detachment (PED) height were 319.7 ± 178.6 µm, 195.5 ± 94.7 µm, and 82.6 ± 115.6 µm, respectively. There were no significant differences in the CCT among the subtypes of nAMD.

**Figure 1:**
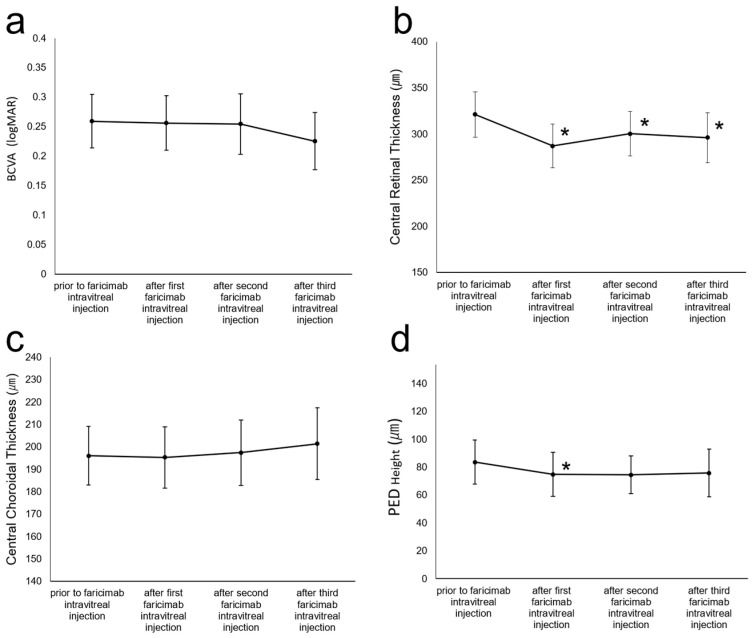
Time course after the first intravitreal injection of faricimab. The vertical bar represents the standard error at each time point. The *p*-values were calculated using the Wilcoxon rank sum test to compare the best-corrected visual acuity (BCVA), central retinal thickness (CRT), central choroidal thickness (CCT), and pigment epithelial detachment (PED) height between baseline and each time point. * *p* < 0.05 compared with the baseline values after the Bonferroni correction. (**a**) Changes in the mean BCVA presented as the logarithm of the minimum angle of resolution (logMAR). (**b**) Changes in the mean CRT (µm). (**c**) Changes in the mean CCT (µm). (**d**) Changes in the mean PED elevation (µm).

Of the 55 patients, 1 patient experienced an ocular adverse event (RPE tear). Figure 1 shows the changes in the BCVA, CRT, CCT, and PED height during follow-up. The mean BCVA did not change at all the time points. Immediately after the initial injection, the CRT significantly decreased, and the reduction was maintained during follow-up (*p* < 0.01 after the first and third injections, *p* = 0.045 after the second injection). Meanwhile, the CCT did not change at all the time points. The PED height significantly decreased after the first IVF (*p* = 0.012). There were 10 eyes without PED elevation before the switch; these eyes did not develop PED elevation during follow-up.

**Figure 2:**
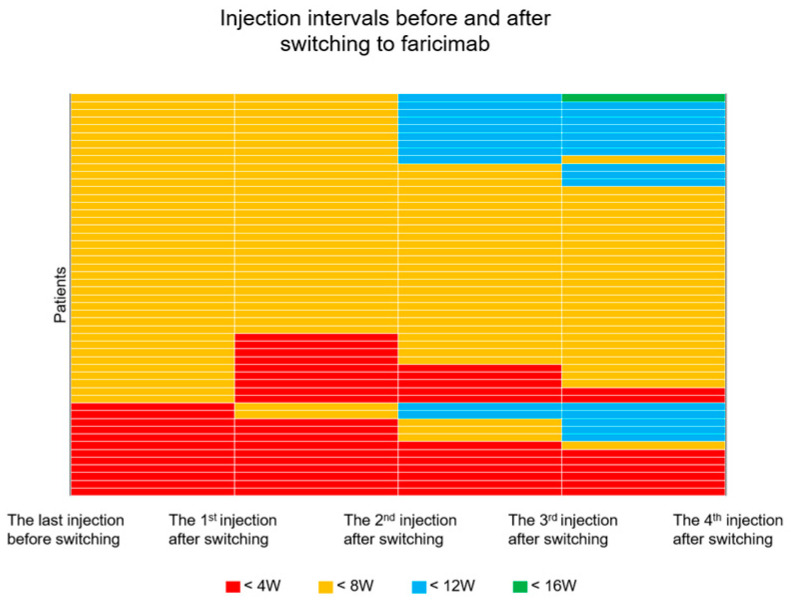
Injection intervals for each patient before and after switching. Patients with exudative changes despite 4 weeks of intravitreal injections are shown in red. Orange, blue, and green represent patients with less than 8, 12, and 16 weeks of intravitreal injections, respectively. The horizontal axis indicates the injection interval after each faricimab injection.

After three injections of faricimab, the interval between injections was significantly extended compared to the IVA before the switch (7.5 ± 2.3 vs. 5.9 ± 1.5 weeks, *p* = 0.004). A treatment interval of ≥8 weeks was achieved in 16 of 55 eyes (29.1%). In 39 of 55 eyes (70.9%), the treatment interval was <8 weeks. The injection intervals for each patient before and after the switch are shown in Figure 2. In some cases, the injection interval could be extended from 4 weeks to 8 weeks or more, while in two cases, the switch shortened the injection interval from <8 weeks to <4 weeks, even with three injections. In those two cases, exacerbations of the exudative changes were observed just before the switch. The treatment duration before the switch was 48 and 84 months, respectively. The number of aflibercept injections before the switch was 23 and 29, respectively. Treatment with the injection interval just before switching did not control disease activity in active disease exacerbations. Therefore, the treatment intervals had to be shortened.

**Figure 3:**
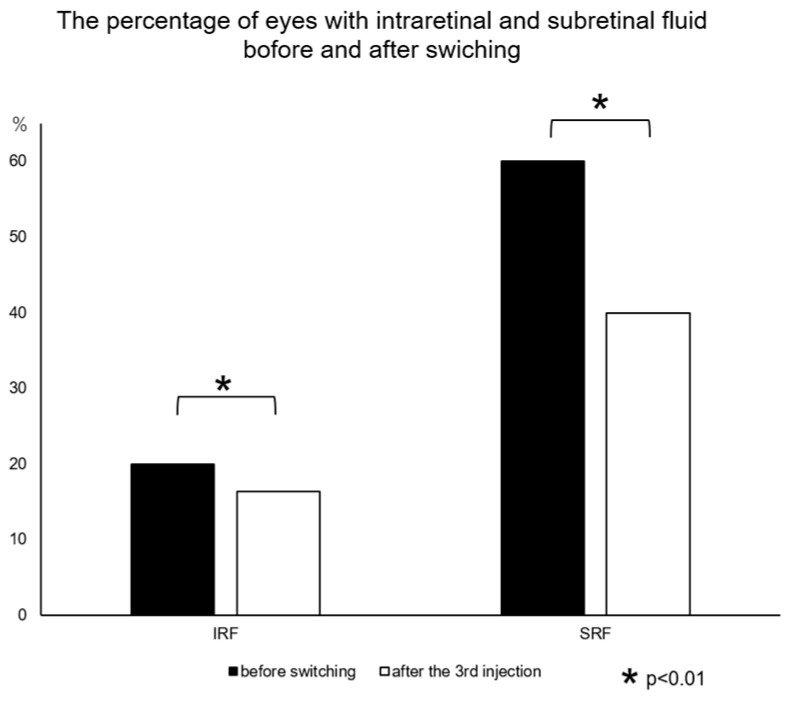
The percentage of eyes with intraretinal and subretinal fluid before and after switching. Significant differences exist in the proportion of eyes with IRF and SRF between before and after three injections of faricimab (both, * *p* < 0.01 after the Bonferroni correction).

Next, we evaluated the presence of IRF and SRF before and after switching to faricimab. Before the switch, IRF was observed in 11 of 55 eyes (20%) and SRF in 33 eyes (60%), and after 3 injections, IRF was observed in 9 of 55 eyes (16.4%) and SRF in 22 eyes (40%), both significant differences (*p* < 0.01) after the Bonferroni correction (Figure 3).

We also examined the characteristics of cases that would benefit from switching. Because clinical practice requires at least an 8-week interval in the maintenance phase, we compared patients with injection intervals of ≥8 weeks and <8 weeks (Table 2). However, no significant differences existed in any of the clinical data prior to switching.

## 4. Discussion

In this study, we investigated the short-term outcomes of faricimab after switching from aflibercept therapy and found that the IVF had an anatomical effect. The treatment outcomes of faricimab in previously treated eyes have been reported in two studies. Rush RB and Rush SW [6] investigated eyes refractory to IVA and compared the visual and anatomical outcomes between the study group (switched from IVA to IVF; 28 eyes) and the control group (continued IVA; 27 eyes). After three injections, 39.3% (11 of 28) of patients in the study group achieved a central macular thickness of <300 microns without retinal fluid compared to 7.4% (2 of 27) of patients in the control group (*p* = 0.004). Additionally, 35.7% (10 of 28) of patients in the study group gained two or more lines of BCVA compared to 7.4% (2 of 27) of patients in the control group (*p* = 0.008). Stanga PE et al. [7] investigated 11 eyes, including 3 treatment-naïve eyes, treated with faricimab. They reported a significant improvement in the BCVA and a decrease in the CRT 1 month after the first injection of faricimab in previously treated eyes. Regarding the CRT, our results were consistent with those of previous reports. However, the visual outcomes did not significantly change after switching to faricimab in this study because of the ceiling effect. The mean BCVA was 0.75 (logMAR) in a report by Rush RB and Rush SW [6] and 0.61 ± 0.75 in a study by Stanga PE et al. [6] In this study, the mean logMAR BCVA was 0.25 ± 0.34. Moreover, there were 51 eyes in which there was no change in visual acuity greater than 0.2 logMAR after switching in the present study. The ellipsoid zone (EZ) integrity of 51 eyes before the switch was the following: absent, 6 eyes; disrupted, 32 eyes; and intact, 13 eyes. In cases where the EZ is absent, changes in visual acuity are less likely to occur. This may be another reason for the visual outcome. Based on data from the TENAYA and LUCERNE studies (phase 3 randomized clinical trials) [4], faricimab demonstrated non-inferiority to aflibercept in terms of the anatomical outcomes in treatment-naïve patients with nAMD. The present study showed that faricimab had an anatomical effect, including the CRT and retinal fluid, even in previously treated eyes that required IVA every <8 weeks. These results suggest that faricimab could be an alternative treatment for some eyes refractory to other treatments.

In the maintenance phase, the financial and physical burden on patients due to frequent treatment is a challenge in relation to treatment adherence. The ALTAIR study (real-world outcomes of TAE of IVA in Japanese patients with nAMD) showed that the proportion of patients with an injection interval of 8 weeks was 41.5% in the 2-week adjustment group and 39.8% in the 4-week adjustment group at 52 weeks. In our study, switching to faricimab significantly extended the injection interval from 5.9 ± 1.5 to 7.5 ± 2.3 weeks (*p* < 0.01), a treatment interval of ≥8 weeks was achieved in 16 of 55 eyes (29.1%), and the proportion of eyes with IRF and SRF was significantly reduced after three injections of faricimab. Moreover, the injection interval could be extended from 4 weeks to 8 weeks or more in some cases. Therefore, faricimab could be considered another treatment option for eyes with a treatment interval of <8 weeks. In this study, we did not find any obvious characteristics of cases that could be extended to injection intervals of ≥8 weeks. The characteristics of patients who would benefit from switching to faricimab should be further investigated with a larger number of cases. On the other hand, the treatment interval was <8 weeks in 39 of 55 eyes (70.9%). In this study, we treated eyes without 4 monthly injections (loading phase), which may have been insufficient for a therapeutic effect. The switch shortened the treatment interval in two cases, even with three injections. In the two cases with shortened injection intervals from <8 weeks to <4 weeks, exacerbations of the exudative changes were observed just before the switch. In particular, in the presence of disease activity, providing 4 monthly injections (loading phase) when switching.

In the present study, one eye developed an ocular adverse event (RPE tear) after a single injection of faricimab. An RPE tear is a complication of intravitreal anti-VEGF injection, with a reported incidence of 1.36% [8]. RPE tears occurred in 2 out of 333 patients (1%) in the TENAYA study and 2 out of 331 patients (1%) in the LUCERNE study at up to 48 weeks after treatment with faricimab. Meanwhile, in the aflibercept groups, an RPE tear did not occur in the TENAYA or LUCERNE studies [4]. The risk factors for an RPE tear after anti-VEGF treatment include PEDs with a height exceeding 400 μm [9] and CNVs with a small ratio compared with the PED [10]. It was reported that the maximum diameter of the PED in the group with an RPE tear was 3.2 mm, which was significantly larger than that in the group without an RPE tear (1.8 mm) [11]. Microrips of the RPE have also been reported as a prognostic sign of the subsequent development of an RPE tear [12]. In the eye that developed an RPE tear in our study, the PED height was 318 μm and the maximum PED diameter was 2508 μm before the switch; no risk factors for the PED size were found on the OCT images. Hence, microrips may have been present because submacular hemorrhage was observed during the switch.

Our study has several limitations, including its retrospective nature, short-term follow-up, and the relatively small number of participants. Therefore, future studies with a larger sample size and a longer follow-up are required.

## 5. Conclusions

In conclusion, switching from aflibercept to faricimab was effective in about a quarter of cases. Faricimab could be an additional treatment option for patients refractory to IVA.

## Figures and Tables

**Table 1:** Patient characteristics.

No. of Eyes	55
Age (yrs), mean ± SD	80.1 ± 7.0
Gender (male/female), no.	34/21
Type of disease (types 1 & 2 MNV/PCV/type 3 MNV), no.	38/16/1
Best-corrected visual acuity prior to faricimab injection (logMAR), mean ± SD	0.26 ± 0.34
Central retinal thickness prior to faricimab injection (μm), mean ± SD	319.7 ± 178.6
Central choroidal thickness prior to faricimab injection (μm), mean ± SD	195.5 ± 94.7
PED elevation prior to faricimab injection (μm), mean ± SD	82.6 ± 115.6
Follow-up duration from the first faricimab injection (weeks), mean ± SD	16.1 ± 5.0
Treatment history before switching to faricimab	
The last treatment interval (weeks), mean ± SD	5.9 ± 1.5
Treatment duration (months), mean ± SD	55.4 ± 44.9
Aflibercept (injections), mean ± SD (range)	23.7 ± 14.8

SD, standard deviation; MNV, macular neovascularization; PCV, polypoidal choroidal vasculopathy; logMAR, logarithm of the minimum angle of resolution; PED, pigment epithelial detachment.

**Table 2:** Comparison between patients with an injection interval of ≥8 weeks and <8 weeks after the third faricimab injection.

	≥8 Weeks (*n* = 16)	<8 Weeks (*n* = 39)	*p* Value
Age, mean ± SD	81.6 ± 6.7	79.5 ± 7.2	0.348
Gender (male/female)	10/6	24/15	0.598
Type of disease (types 1 & 2 MNV/PCV/type 3 MNV), mean ± SD	12/4/0	25/13/1	0.649
Best-corrected visual acuity prior to faricimab injection (logMAR), mean ± SD	0.23 ± 0.37	0.27 ± 0.33	0.455
Central retinal thickness prior to faricimab injection (µm), mean ± SD	312.2 ± 188.9	325.0 ± 180.9	0.470
Central choroidal thickness prior to faricimab injection (µm), mean ± SD	223.7 ± 88.8	184.8 ± 98.1	0.120
PED elevation prior to faricimab injection (µm), mean ± SD	98.5 ± 164.4	77.5 ± 93.8	0.353
The last treatment interval prior to faricimab injection (weeks), mean ± SD	6.1 ± 1.7	5.7 ± 1.4	0.486
Treatment duration prior to faricimab injection (months), mean ± SD	49.6 ± 39.3	59.7 ± 47.3	0.499
Number of aflibercept injections prior to faricimab injection, mean ± SD	21.4 ± 13.7	24.5 ± 15.3	0.321

SD, standard deviation; MNV, macular neovascularization; PCV, polypoidal choroidal vasculopathy; logMAR, logarithm of the minimum angle of resolution; PED, pigment epithelial detachment.

## Data Availability

Not applicable.
